# Outcomes in Patients With Resectable Stage III NSCLC Who Did Not Have Definitive Surgery After Neoadjuvant Treatment—A Retrospective Analysis of the SAKK Trials 16/96, 16/00, 16/01, 16/08, and 16/14: A Brief Report

**DOI:** 10.1016/j.jtocrr.2025.100834

**Published:** 2025-04-09

**Authors:** Sabine Raimann, Sämi Schär, Stefanie Hayoz, Matthias Guckenberger, Tobias Finazzi, Isabelle Opitz, Sabine Schmid, Michael Mark, Alfredo Addeo, Laetitia A. Mauti, Daniel C. Betticher, Hans-Beat Ris, Roger Stupp, Alessandra Curioni-Fontecedro, Solange Peters, Martin Früh, Sacha I. Rothschild, Miklos Pless, David König

**Affiliations:** a Department of Medical Oncology, Cantonal Hospital of Winterthur, Winterthur, Switzerland; b University of Basel, Basel, Switzerland; c Swiss Group for Clinical Cancer Research (SAKK), Bern, Switzerland; d Department of Radiation Oncology, University Hospital Zurich, University of Zurich, Zurich, Switzerland; e Clinic of Radiotherapy and Radiation Oncology, University Hospital of Basel, Basel, Switzerland; f Department of Radiation Oncology, Cantonal Hospital Baden, Baden, Switzerland; g Department of Thoracic Surgery, University Hospital of Zurich, Zurich, Switzerland; h Department of Medical Oncology, University Hospital of Bern (Inselspital), Bern, Switzerland; i Department of Oncology, Cantonal Hospital of Graubünden, Chur, Switzerland; j Università della Svizzera Italiana, Lugano, Switzerland; k Department of Oncology, University Hospital of Geneva, Geneva, Switzerland; l Clinic of Medical Oncology, Cantonal Hospital of Fribourg (HFR), Fribourg, Switzerland; m Clinic for Thoracic Surgery, Hôpital du Valais, Sion, Switzerland; n Lurie Comprehensive Cancer Center, Northwestern University Feinberg School of Medicine, Illinois; o Department of Oncology, University Hospital of Lausanne (CHUV), Lausanne, Switzerland; p University of Fribourg, Fribourg, Switzerland; q Department of Medical Oncology/Hematology, Cantonal Hospital of St. Gallen, St. Gallen, Switzerland; r Center for Medical Oncology and Hematology, Cantonal Hospital Baden, Baden, Switzerland; s Department of Medical Oncology, University Hospital of Basel, Basel, Switzerland

**Keywords:** Resectable NSCLC, Neoadjuvant treatment, Definitive surgery, Progression, Salvage treatment

## Abstract

**Introduction:**

Neoadjuvant or perioperative treatment, including an immune checkpoint inhibitor (ICI), has emerged as a new standard for patients with resectable stage III NSCLC. Nevertheless, approximately 20% of patients who start neoadjuvant chemo-immunotherapy will not undergo definitive surgery. Little is known about these patients.

**Methods:**

We analyzed outcomes of patients without definitive surgery from five Swiss Group for Clinical Cancer Research (SAKK) trials that investigated different neoadjuvant treatment modalities in patients with resectable stage III-N2 NSCLC. Study treatment included neoadjuvant cisplatin-docetaxel chemotherapy (with or without radiotherapy), either combined with peri-operative durvalumab in the SAKK 16/14 trial (n = 68) or without an ICI (non-ICI trials, n = 431).

**Results:**

Of the 499 patients, 102 (20%) did not have definitive surgery. Cancellation of surgery occurred in a similar proportion of patients with or without neoadjuvant ICI (19% versus 21%, *p* = 0.9). Reasons were in non-ICI trials and SAKK 16/14: disease progression (47% and 54%), nonresectability (18% and 8%), medical reasons (17% and 31%), and unknown (18% and 8%), respectively. Of these patients, no patient in SAKK 16/14 and 17 patients (19%) in the non-ICI trials received curative-intended salvage therapy. Three-year overall survival was higher in patients who had definitive surgery compared with those who did not: 78% versus 32% (SAKK 16/14) and 54% versus 10% (non-ICI trials).

**Conclusions:**

In our pooled analysis, patients with definitive surgery had higher survival rates than those without definitive surgery. Prognosis in patients without definitive surgery seems to have improved in the era of ICI.

## Introduction

Several phase 3 trials support the incorporation of immune checkpoint inhibitors (ICIs) targeting the programmed cell death protein-1 or programmed death-ligand 1 axis in the neoadjuvant or perioperative treatment of resectable NSCLC.^1^, ^2^, ^3^, ^4^ There are various reasons in favor of a neoadjuvant over an adjuvant-only administration of ICI, including mechanistic aspects.^5^ Nevertheless, a concern of a neoadjuvant treatment is the possible delay and even missed opportunity for definitive surgery owing to treatment-associated toxicities or early disease progression (PD). In the pivotal phase 3 trials, the rate of randomized patients who did not undergo definitive surgery, defined as completed surgery with curative intent, was 18% to 23% in the ICI-containing treatment arms and 23% to 27% in the chemotherapy-only arms.^1^, ^2^, ^3^, ^4^ Although reasons for not undergoing surgery have been well described in the corresponding studies, details on the treatment and outcome of patients without definitive surgery have been scarcely reported. The Swiss Group for Clinical Cancer Research (SAKK) has been investigating the treatment for resectable stage III NSCLC in five consecutive trials since 1996, all built on a backbone of cisplatin-docetaxel chemotherapy (SAKK trials 16/96, 16/00, 16/01, 16/08, and 16/14 [with 16 denoting the disease site and the second number the trial’s start year]), whereas the protocol of the SAKK 16/14 study also investigated the neoadjuvant addition of immunotherapy.^6^, ^7^, ^8^, ^9^, ^10^

Herein, we report an updated pooled analysis from these five SAKK trials with a focus on enrolled patients who ultimately did not undergo definitive surgical resection.

## Materials and Methods

The detailed trial designs, inclusion and exclusion criteria, and methods of the SAKK trials have been previously published.^6^, ^7^, ^8^, ^9^, ^10^ The trials were conducted sequentially between 1997 and 2019. It is important to note that the inclusion criteria differed across the SAKK trials. Although SAKK 16/01 and 16/08 included patients with T4 N0-3 or T1-4 N3, the SAKK 16/96, 16/00, and 16/14 trials enrolled T1-3 N2 patients only. Eligibility and operability across the trials were assessed by a multidisciplinary tumor board. Patients were pathologically staged with mediastinoscopy or (once available) with endobronchial ultrasound. Imaging studies included computed tomography (CT) scans of the chest and abdomen and magnetic resonance imaging of the brain. Only 62% of patients from the SAKK 16/96, 16/00, 16/01, and 16/08 had staging with ^18^F-fluorodeoxyglucose (FDG) positron emission tomography (PET)–CT (PET-CT), as introduced in clinical practice in the early 2000s. All patients were previously untreated and received neoadjuvant cisplatin-docetaxel chemotherapy (cisplatin 80–100 mg/m^2^ and docetaxel 85 mg/m^2^, every 3 weeks for three cycles), followed in some patients by neoadjuvant sequential radiotherapy (44 Gy in 22 fractions over 3 wk) (SAKK 16/00, 16/01, and 16/08) and cetuximab (weekly for 12 wk) (SAKK 16/08). Patients in the SAKK trial 16/14 received two doses of the programmed death-ligand 1 inhibitor durvalumab (750 mg once every 2 wk) preoperatively and no radiotherapy. Surgery included an anatomical tumor resection with mediastinal lymph node dissection. Patients in SAKK 16/14 continued postoperative durvalumab (750 mg once every 2 wk) for the duration of 1 year. We pooled data from patients in the SAKK trials that did not include the administration of an ICI (SAKK 16/96, 16/00, 16/01, and 16/08). We then converted the fifth (16/96) and sixth (16/00, 16/01, and 16/08) editions of the TNM staging system to the seventh edition, as used in the SAKK 16/14 trial, for the current analysis. Response and follow-up assessments for each of the studies were carried out according to the specific study protocol. Definitive surgery was defined as surgery with complete resection. Study data have been updated (data cutoff: January 17, 2024). All SAKK trials were done in accordance with the Declaration of Helsinki and the guidelines on good clinical practice. The protocols were approved by local ethics committees. Written informed consent was obtained from all patients. Approval for this retrospective analysis was obtained by the Ethics Committee of Northwestern and Central Switzerland (EKNZ, Switzerland, BASEC-Nr. 2024-00281). Statistical analyses included frequencies and percentages for categorical data and the median and range for continuous data. For categorical variables, Fisher’s exact test was used in subgroup analyses. For time-to-event end points, Kaplan-Meier estimates and corresponding 95% confidence intervals on the basis of the log-log approach were used to describe and visualize the effect of categorical variables. Survival curves and rates between groups were compared using the log-rank test and the Kaplan-Meier method, respectively at a given time point. All analyses were performed using R version 4.2.3 (http://www.r-project.org).

## Results

A total of 499 patients were included in this analysis, 431 patients from non-ICI SAKK trials and 68 patients from SAKK 16/14. Patient demographics and disease characteristics are summarized in Supplementary Table 1. Overall, 102 patients (20%) did not undergo definitive surgery. Patients without definitive surgery were more likely to have T4 (21% versus 14%, *p* = 0.128) or stage IIIB disease (19% versus 15%, *p* = 0.353) than patients with definitive surgery. The highest rate of patients with cancellation of surgery was reported for the SAKK trial 16/01 (35%), likely related to the study population, which included patients with resectable stage IIIB only.

**Figure 1:**
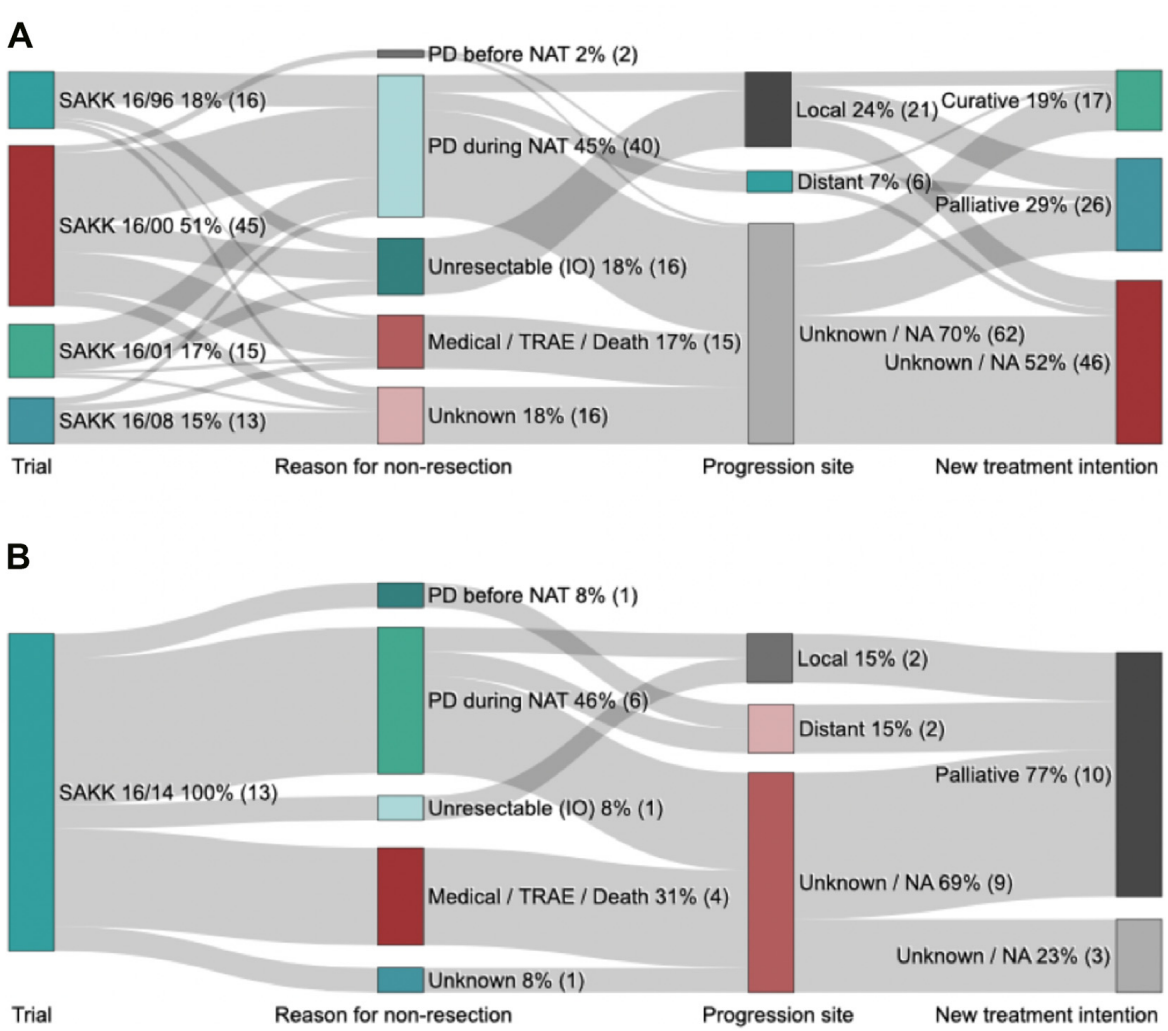
Reason for not undergoing definitive surgery, subsequent treatment, and treatment intention in the 89 patients from non-ICI trials *(A)* and in the 13 patients from SAKK 16/14 *(B)*. ICI, immune checkpoint inhibitor; IO, intraoperative; NA, not applicable; NAT, neoadjuvant treatment; PD, progression disease; SAKK, Swiss Group for Clinical Cancer Research; TRAE, treatment-related adverse event.

In the non-ICI trials, reasons for not having definitive surgery (n = 89) were PD during neoadjuvant treatment or before (n = 42, 47%), intraoperative diagnosis of nonresectability (n = 16, 18%), medical reasons including death without documented progression and treatment related toxicity (n = 15, 17%) and remained unknown in 16 patients (18%) (Fig. 1*A*). Of all patients with PD (n = 42), loco-regional progression occurred in five (12%), distant progression in six (14%), and the site of progression was unknown in 31 patients (74%). Curative-intent treatment at PD was reported in 17 patients (19%) with a median overall survival (OS) of 18.4 months, 3- and 5-year OS of 35% and 29%, respectively. Of 13 patients from SAKK 16/14 without definitive surgery, seven (54%) had PD, one (8%) was considered unresectable intraoperatively, and medical reasons were reported in four patients (31%), no information was found in one patient (8%) (Fig. 1*B*). No salvage therapy was reported. Details including type of study treatment, reasons for not undergoing definitive surgery, site of progression, and subsequent therapies are summarized in Supplementary Tables 2 and 3.

Among patients who underwent definitive surgery, R0 resection was achieved in more patients after neoadjuvant ICI-based treatment (SAKK 16/14) compared with the non-ICI trials (93% versus 80%, *p* = 0.024).

**Figure 2:**
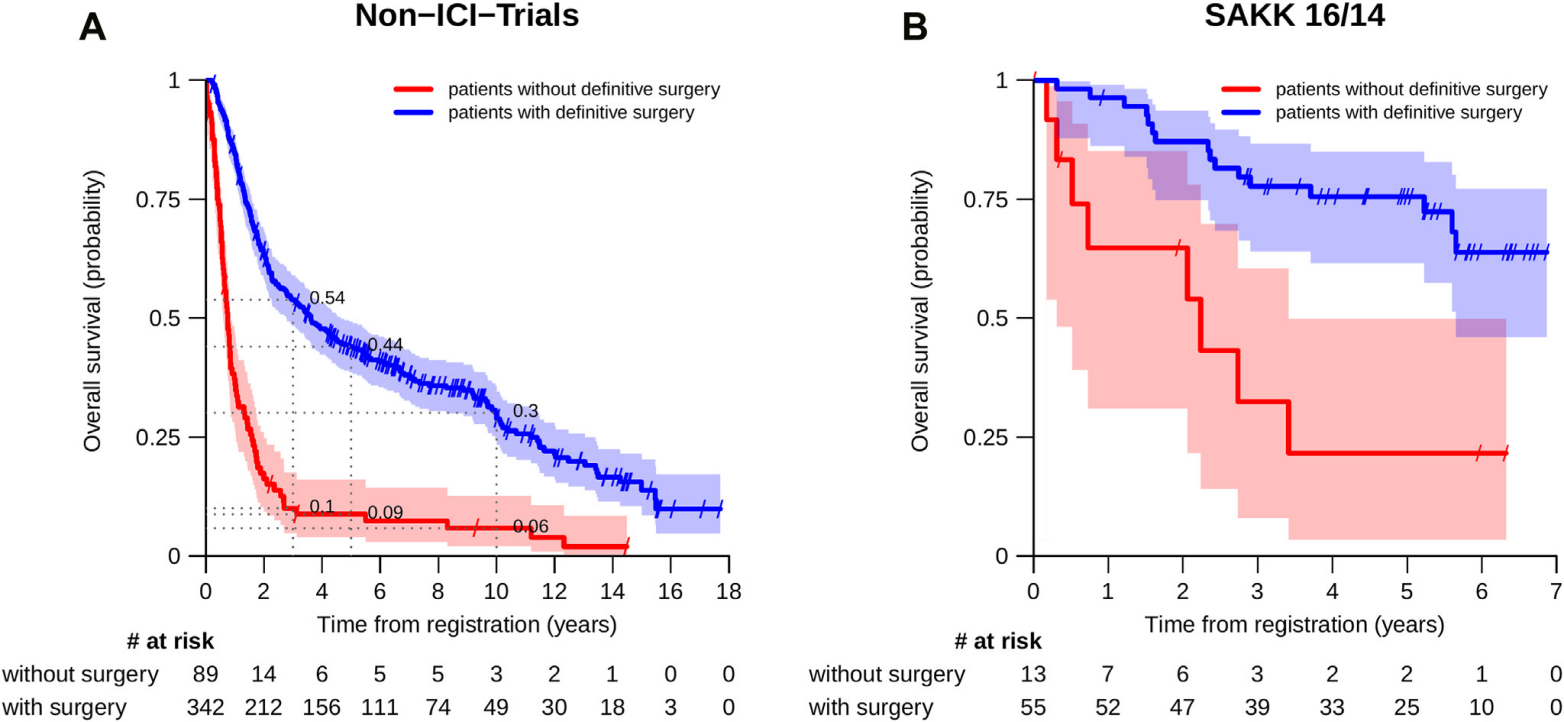
Overall survival in patients with and without definitive surgery in the non-ICI trials (SAKK 16/96, 16/00, 16/01, and 16/08) *(A)* compared with the SAKK 16/14 trial *(B)*. ICI, immune checkpoint inhibitor; SAKK, Swiss Group for Clinical Cancer Research.

With a median follow-up of 9.5 years, the 3- and 5-year OS rates in the non-ICI trials were 45% and 37%. In SAKK 16/14 with a median follow-up of 4.5 years, the 3-year OS rate was 70% (Supplementary Fig. 1*A* and *B*). The median OS was longer in patients who had definitive surgery than in those who did not (Fig. 2*A* and *B*). In an explorative comparison between the early trials (non-ICI trials) and the SAKK 16/14, cancellation of surgery occurred in a similar proportion of patients (19% in SAKK 16/14 versus 21% in non-ICI trials, *p* = 0.9). Interestingly, 3- and 5-year OS rates for patients without definitive surgery are considerably higher for patients from SAKK 16/14 compared with those from the non-ICI trials (Table 1). The median OS according to resection status in the non-ICI trials and the SAKK trial 16/14 are reported in Supplementary Figure 2*A* and *B*. Subsequent treatment in patients who did not undergo definitive surgery was associated with improved median OS compared with no subsequent treatment, regardless of the treatment intent (Supplementary Fig. 3).

**Table 1:** Overall Survival (Median; 3-, 5- and 10-Y OS) According to SAKK Trial and Definitive Versus No Definitive Surgery

Trial		Non-ICI SAKK trials^a^	SAKK 16/96	SAKK 16/00	SAKK 16/01	SAKK 16/08	SAKK 16/14
Intention to treat	mOS^b^	27.2 (22.8–35.1)	27.4 (16.6–39.0)	35.4 (25.2–50.0)	28.7 (14.0–65.8)	21.5 (13.7–25.4)	NR (67.2–NR)
	3-, 5-, 10-y OS^c^	45 (40–50) 37 (32–41) 25 (21–30)	40 (30–50) 33 (24–43) 20 (12–30)	50 (43–56) 40 (34–46) 28 (21–35)	47 (31–60) 37 (23–51) 25 (13–39)	35 (24–46) 31 (21–43) NA	71 (58–80) 67 (54–77) NA
Definitive surgery	mOS^b^	43.4 (33.3–55.7)	33 (21.8–0.6)	51.5 (37.9–80.1)	73.3 (33.3–122.5)	24 (18.8–40.7)	NR (67.8–NR)
	3-, 5-, 10-y OS^c^	54 (48–59) 44 (39–49) 30 (25–36)	48 (36–59) 39 (28–50) 23 (14–35)	58 (51–65) 47 (40–54) 33 (25–40)	68 (47–82) 54 (34–70) 39 (21–56)	39 (27–52) 36 (23–48) NA	78 (64–87) 76 (62–85) NA
No definitive Surgery	mOS^b^	9 (7.1–10.2)	9 (5.9–13.4)	9.5 (6.7–12.6)	8.8 (3.8–10.1)	7.6 (4.4–22.5)	26.8 (3.7–40.9)
	3-, 5-, 10-y OS^c^	10 (4.8–18) 8.8 (4.0–16) 5.9 (2.1–13)	6.3 (0.4–25) 6.3 (0.4–25) 6.3 (0.4–25)	12 (4.4–23) 9.5 (3.0–20) 7.1 (1.8–17)	6.7 (0.4–26) 6.7 (0.4–26) NA	15 (2.5–39) NA NA	32 (8–60) 22 (3.4–50) NA

CI, confidence interval; ICI, immune checkpoint inhibitor; mOS, median overall survival; NA, not applicable; NR, not reached; OS, overall survival; SAKK, Swiss Group for Clinical Cancer Research.

a Pooled data of SAKK 16/96, SAKK 16/00, SAKK 16/01, and SAKK 16/08.

b Months (95% CI)

c % (95% CI)

## Discussion

In this retrospective analysis of five SAKK trials for resectable stage III NSCLC, we analyzed patterns of progression, subsequent treatments, and outcomes in 102 patients who did not have definitive surgery. The rate of cancellation of surgery of 20% (21% in earlier non-ICI trials, 19% in SAKK 16/14) is very similar to the corresponding rates in recently published phase 3 trials that investigated neoadjuvant or perioperative chemo-immunotherapy. Importantly, the SAKK study population included stage III patients only, mostly patients with N2 disease (84%). It is also important to note that imaging and invasive staging have improved significantly over the last 30 years, with possible stage migration in the earlier SAKK trials.

In our analysis, the most common reasons for cancellation of surgery were PD before or during neoadjuvant treatment and intraoperative unresectability. The rate seems to be slightly lower in the latest SAKK 16/14 trial (54%) than in the earlier SAKK trials (64%), which may be due to different reasons, such as surgical advances, and cannot necessarily be attributed to the additional benefit of an ICI. Salvage treatment with a curative intent for patients who did not undergo definitive surgery was given to 17 patients (19%) in the non-ICI trials, with 3- and 5-year OS of 35% and 29%, respectively. In the SAKK 16/14, 13 patients remained without definitive surgery, and none received salvage treatment. Of these, seven patients (53%) received palliative immunotherapy, which was not offered in any of the other trials. Owing to the retrospective data collection for this analysis (with missing information), it cannot be excluded that more patients underwent salvage treatment. Nevertheless, this confirms that curative-intended procedures, mainly chemoradiotherapy, remain possible for some, though only a minority of patients. Interestingly, the proportion of patients receiving definitive chemoradiotherapy was surprisingly low. It is important to note that some patients had previously received chemoradiotherapy as part of the study treatment, thereby precluding its use as a rescue treatment. Furthermore, it advocates for a structured follow-up of these patients to identify those who may be eligible for such a procedure.

We report higher survival rates in patients who had definitive surgery compared with patients without definitive surgery. This observation is in line with findings from various other trials with a neoadjuvant treatment approach, both with ICI and non-ICI containing neoadjuvant treatment regimens. Our analysis shows that the prognosis of patients who did not undergo surgery is poor, compared with those who did. This observation is valid for every single SAKK study. It is noteworthy that the 3- and 5-year survival rates in patients without definitive surgery in the most recent ICI-containing SAKK 16/14 trial were 32% and 22%, respectively, whereas in the other SAKK trials, this rate was much lower (Table 1), suggesting possible improvement of the prognosis in these patients. The introduction of new systemic treatment options in the palliative setting, such as ICIs and targeted therapies, may have significantly enhanced survival outcomes in patients who do not undergo definitive surgery. From the 13 patients in the SAKK 16/14 trial who did not undergo definitive surgery, seven received subsequent ICI-containing treatments, and one patient had targeted treatment with a tyrosine kinase inhibitor. In addition, radiotherapy options, such as local ablative radiotherapy of progressive metastases, have improved significantly in recent years. Of the 13 patients from SAKK 16/14 mentioned above, five have received subsequent radiotherapy in some form. It is also important to mention that staging methods have improved significantly over the last 30 years (FDG PET-CT, endobronchial ultrasound), allowing for better patient selection.

Our analysis has several strengths and limitations. This pooled data analysis combines a large, homogeneous study population of patients with resectable stage III NSCLC treated in a multicentric setting over more than two decades using the same neoadjuvant chemotherapy backbone, allowing for focus on those patients who did not undergo definitive surgery. Cross-trial comparison between the single SAKK trials is not possible owing to multiple reasons, including slight discrepancies in patient selection criteria, differences in staging (no FDG PET-CT and endobronchial ultrasound available in the early SAKK trials), and advancements in surgical techniques over the years. A limitation of the current analysis is the lack of information regarding subsequent therapies (agents, duration, and response) in many patients who did not undergo surgery. This is owing to the retrospective nature of the present analysis and the lack of structured recording of parameters of interest in the early SAKK studies.

Our analysis confirmed the known reasons for not having definitive surgery in patients with resectable stage III NSCLC and the poor prognosis. Yet, our analysis also suggests that the prognosis in this group of patients seems to have improved in recent times. We found that a curative-intent treatment approach can be performed in a proportion of these patients, although a minority.

## CRediT Authorship Contribution Statement

**Sabine Raimann:** Investigation, Resources, Data curation, Visualization, Writing – original draft, Writing - review & editing, Project administration, Final approval.

**Sämi Schär:** Methodology, Formal analysis, Resources, Data curation, Visualization, Writing - original draft, Writing - review & editing, Final approval.

**Stefanie Hayoz:** Methodology, Formal analysis, Resources, Data curation, Visualization, Writing - original draft, Writing - review & editing, Final approval.

**Matthias Guckenberger:** Data curation, Resources, Writing - review & editing, Final approval.

**Tobias Finazzi:** Data curation, Resources, Writing - review & editing, Final approval.

**Isabelle Opitz:** Data curation, Resources, Writing - review & editing, Final approval.

**Sabine Schmid:** Data curation, Resources, Writing - review & editing, Final approval.

**Michael Mark:** Data curation, Resources, Writing - review & editing, Final approval.

**Alfredo Addeo:** Data curation, Resources, Writing - review & editing, Final approval.

**Laetitia A. Mauti:** Data curation, Resources, Writing - review & editing, Final approval.

**Daniel C. Betticher:** Data curation, Resources, Writing - review & editing, Final approval.

**Hans-Beat Ris:** Data curation, Resources, Writing - review & editing, Final approval.

**Roger Stupp:** Data curation, Resources, Writing - review & editing, Final approval.

**Alessandra Curioni-Fontecedro:** Data curation, Resources, Writing - review & editing, Final approval.

**Solange Peters:** Data curation, Resources, Writing - review & editing, Final approval.

**Martin Früh:** Data curation, Resources, Writing - review & editing, Final approval.

**Sacha I. Rothschild:** Data curation, Resources, Writing - review & editing, Final approval.

**Miklos Pless:** Conceptualization, Methodology, Investigation, Data curation, Resources, Writing - original draft, Writing - review & editing, Final approval.

**David König:** Conceptualization, Methodology, Investigation, Data curation, Resources, Visualization, Writing - original draft, Writing - review & editing, Supervision, Project administration, Funding acquisition, Final approval.

## Disclosure

Dr. Raimann received travel support from 10.13039/100015756Janssen-Cilag, Sanofi, and BeiGene. Dr. Finazzi received honoraria for lectures, presentations, or speakers’ bureaus from AstraZeneca; travel support from 10.13039/100004325AstraZeneca, Astellas, and Debiopharm. Dr. Opitz received honoraria for lectures, presentations, or speakers’ bureaus from AstraZeneca and Roche; and holds positions on advisory boards from AstraZeneca, Merck Sharp & Dohme, Medtronic, and Bristol-Myers Squibb; other financial or nonfinancial interests include Roche (Intuitive - Proctorship) and Medtronic. Dr. Mark received consulting fees from Janssen, Roche, Takeda, Bristol-Myers Squibb, Merck Sharp & Dohme, AstraZeneca, and Merck; and travel support from Janssen, AstraZeneca, Roche, Takeda, and Amgen. Dr. Addeo received grants from AstraZeneca; honoraria for lectures, presentations, or speakers’ bureaus from AstraZeneca, Eli Lilly, and Amgen; and holds positions on advisory boards from Bristol-Myers Squibb, AstraZeneca, Roche, Astellas, Novartis, Merck Sharp & Dohme, Pfizer, Eli Lilly, Amgen, and Merck. Dr. Mauti received grants from AstraZeneca and Gilead; honoraria for lectures, presentations or speakers’ bureaus from Amgen; travel support from AstraZeneca, Roche and Sanofi; and holds positions on advisory boards from Takeda, Bristol-Myers Squibb, Merck Sharp & Dohme, Merck, Sanofi, Novartis, AstraZeneca, Pfizer, Regeneron, Daiichi Sankyo and Sanofi. Dr. Früh received consulting fees (all to institution) from Bristol-Myers Squibb, Merck Sharp & Dohme, AstraZeneca, Boehringer Ingelheim, Roche, Takeda, Pfizer, Janssen, Daiichi Sankyo, and PharmaMar. Dr. Rothschild holds research grants from AstraZeneca, Merck, Serono, Roche and Amgen; received honoraria for lectures, presentations or speakers bureaus from Roche, Bristol-Myers Squibb, AstraZeneca, Amgen, Merck Sharp & Dohme, Novartis, Roche Diagnostics and Takeda; honoraria for expert testimony from Roche, AstraZeneca and Bristol-Myers Squibb; travel support from 10.13039/100004337Roche, Eli Lilly, 10.13039/100002491Bristol-Myers Squibb, Amgen, AstraZeneca and Merck Sharp & Dohme; and holds positions on advisory boards from Amgen, AstraZeneca, Bayer, Bristol-Myers Squibb, Boehringer Ingelheim, Eli Lilly, Janssen, Merck, Merck Sharp & Dohme, Novartis, Otsuka, Pfizer, PharmaMar, Roche Pharma, Bristol-Myers Squibb and Takeda; other nonfinancial interests include Member/Vice-President from the Swiss Group for Clinical Cancer Research (SAKK) and Member (elected Member) from the Swiss Federal Drug Commission (Federal Health Office). Dr. Pless received consulting fees from AbbVie, AstraZeneca, Bristol-Myers Squibb, Roche, Takeda, Eisai Pharma, Merck Sharp & Dohme, Novartis, Pfizer and Merck; honoraria for lectures, presentations or speakers’ bureaus from Janssen, Bayer, Nestlé, Sanofi and Amgen; travel support from AstraZeneca, Bristol-Myers Squibb, Boehringer Ingelheim, Roche, Takeda and Vifor. Dr. König received institutional grants from Geistlich-Stucki Stiftung; consulting fees from AstraZeneca, Merck Sharp & Dohme und Novartis; honoraria for lectures, presentations or speakers’ bureaus from Amgen, SanofiSanofi, Swiss Oncology in Motion and Mirati; travel support from 10.13039/100004339Sanofi, 10.13039/100002429Amgen and Roche; and holds positions on advisory boards from AstraZeneca, PharmaMar, Merck Bristol-Myers Squibb and Merck Sharp & Dohme. The remaining authors declare no conflict of interest.
