# Supplementary material for: Outcomes in Patients With Resectable Stage III NSCLC Who Did Not Have Definitive Surgery After Neoadjuvant Treatment—A Retrospective Analysis of the SAKK Trials 16/96, 16/00, 16/01, 16/08, and 16/14: A Brief Report
Source: JTO Clin Res Rep. 2025 Apr 9;6(6):100834. doi: 10.1016/j.jtocrr.2025.100834 (PMC12104641; doi:10.1016/j.jtocrr.2025.100834)

**Supplementary material**

**Table S1**. Patient’s demographics and disease characteristics.

Cohort A (n=102) includes patients without definitive surgery. Cohort B (n=397) includes patients with definitive surgery. Cohort B1 (n=323) includes patients with R0 resection and Cohort B2 (n=74) patients with R1/R2 resection.

|  |  | **Cohort** | | | | | |
| --- | --- | --- | --- | --- | --- | --- | --- |
| **Characteristic** | **Overall**, n=499^1^ | | **A**, n=102^1^ | **B**, n=397^1^ | **p-value^2^**, A vs. B | **B1**, n=323^1^ | **B2**, n=74^1^ |
| Trial |  | |  |  | 0.2 |  |  |
| SAKK 16/96 | 88 (18%) | | 16 (16%) | 72 (18%) |  | 40 (12%) | 32 (43%) |
| SAKK 16/00 | 231 (46%) | | 45 (44%) | 186 (47%) |  | 165 (51%) | 21 (28%) |
| SAKK 16/01 | 43 (8.6%) | | 15 (15%) | 28 (7.1%) |  | 25 (7.7%) | 3 (4.1%) |
| SAKK 16/08 | 69 (14%) | | 13 (13%) | 56 (14%) |  | 42 (13%) | 14 (19%) |
| SAKK 16/14 | 68 (14%) | | 13 (13%) | 55 (14%) |  | 51 (16%) | 4 (5.4%) |
| Age at registration | 60 (28, 76) | | 61 (28, 76) | 60 (30, 76) | 0.077 | 60 (30, 76) | 58 (36, 73) |
| Gender |  | |  |  | 0.8 |  |  |
| Female | 158 (32%) | | 31 (30%) | 127 (32%) |  | 109 (34%) | 18 (24%) |
| Male | 341 (68%) | | 71 (70%) | 270 (68%) |  | 214 (66%) | 56 (76%) |
| ECOG Performance status |  | |  |  | 0.2 |  |  |
| 0 | 330 (66%) | | 57 (56%) | 273 (69%) |  | 218 (67%) | 55 (74%) |
| 1 | 157 (31%) | | 38 (37%) | 119 (30%) |  | 102 (32%) | 17 (23%) |
| 2 | 1 (0.2%) | | 0 (0%) | 1 (0.3%) |  | 0 (0%) | 1 (1.4%) |
| (Missing) | 11 (2.2%) | | 7 (6.9%) | 4 (1.0%) |  | 3 (0.9%) | 1 (1.4%) |
| Histology |  | |  |  | 0.011 |  |  |
| Squamous | 180 (36%) | | 29 (28%) | 151 (38%) |  | 122 (38%) | 29 (39%) |
| Adenocarcinoma | 203 (41%) | | 40 (39%) | 163 (41%) |  | 138 (43%) | 25 (34%) |
| Large cell | 34 (6.8%) | | 12 (12%) | 22 (5.6%) |  | 13 (4.0%) | 9 (12%) |
| Poorly differentiated NSCLC | 71 (14%) | | 16 (16%) | 55 (14%) |  | 46 (14%) | 9 (12%) |
| Not otherwise specified | 9 (1.8%) | | 5 (4.9%) | 4 (1.0%) |  | 2 (0.6%) | 2 (2.7%) |
| TNM T (7^th^ Edition) |  | |  |  | 0.4 |  |  |
| T1 | 66 (13%) | | 12 (12%) | 54 (14%) |  | 50 (15%) | 4 (5.4%) |
| T2 | 225 (45%) | | 42 (41%) | 183 (46%) |  | 143 (44%) | 40 (54%) |
| T3 | 130 (26%) | | 27 (26%) | 103 (26%) |  | 83 (26%) | 20 (27%) |
| T4 | 78 (16%) | | 21 (21%) | 57 (14%) |  | 47 (15%) | 10 (14%) |
| TNM N (7^th^ Edition) |  | |  |  | 0.082 |  |  |
| N0 | 32 (6.4%) | | 12 (12%) | 20 (5.0%) |  | 15 (4.6%) | 5 (6.8%) |
| N1 | 5 (1.0%) | | 0 (0%) | 5 (1.3%) |  | 5 (1.5%) | 0 (0%) |
| N2 | 417 (84%) | | 81 (79%) | 336 (85%) |  | 275 (85%) | 61 (82%) |
| N3 | 45 (9.0%) | | 9 (8.8%) | 36 (9.1%) |  | 28 (8.7%) | 8 (11%) |
| TNM M (7^th^ Edition) |  | |  |  | NA |  |  |
| M0 | 499 (100%) | | 102 (100%) | 397 (100%) |  | 323 (100%) | 74 (100%) |
| UICC stage |  | |  |  | 0.4 |  |  |
| IIIA | 354 (71%) | | 70 (69%) | 284 (72%) |  | 227 (70%) | 57 (77%) |
| IIIB | 77 (15%) | | 19 (19%) | 58 (15%) |  | 45 (14%) | 13 (18%) |
| (Missing) | 68 (14%) | | 13 (13%) | 55 (14%) |  | 51 (16%) | 4 (5.4%) |
| Smoking status |  | |  |  | 0.2 |  |  |
| Current | 264 (53%) | | 62 (61%) | 202 (51%) |  | 169 (52%) | 33 (45%) |
| Former | 199 (40%) | | 33 (32%) | 166 (42%) |  | 133 (41%) | 33 (45%) |
| Never | 36 (7.2%) | | 7 (6.9%) | 29 (7.3%) |  | 21 (6.5%) | 8 (11%) |
| Pack years | 45 (0, 182) | | 49 (0, 140) | 45 (3, 182) | >0.9 | 45 (3, 182) | 45 (10, 100) |
| Unknown | 48 | | 8 | 40 |  | 28 | 12 |
| ^1^n (%); ^2^Pearson’s Chi-squared test, Wilcoxon rank sum test, Fisher’s exact test; Median (Range); ECOG Eastern Cooperative Oncology Group; TNM according to the 7th edition of AJCC. | | | | | | | |

**Table S2.** Study treatment received, reasons for not undergoing definitive surgery, site of disease progression (if applicable), subsequent treatment, and subsequent treatment intent in non-ICI clinical trials (SAKK 16/96, -SAKK 16/00, SAKK 16/01 and SAKK 16/08 trials). The first column indicates the name of the specific SAKK trial. The second column (neoadjuvant chemotherapy, NA-CHT) describes whether the patient received neoadjuvant chemotherapy or not. The third column (neoadjuvant radiotherapy, NA-RT) describes whether the patient received neoadjuvant radiotherapy or not or if radiotherapy was not part of the study procedure (Not applicable). The fourth column summarizes the reasons for not undergoing definitive surgery (non-resection). The fifth column summarizes the site of progression (if known). The sixth column summarizes subsequent treatment following non-resection (if known).


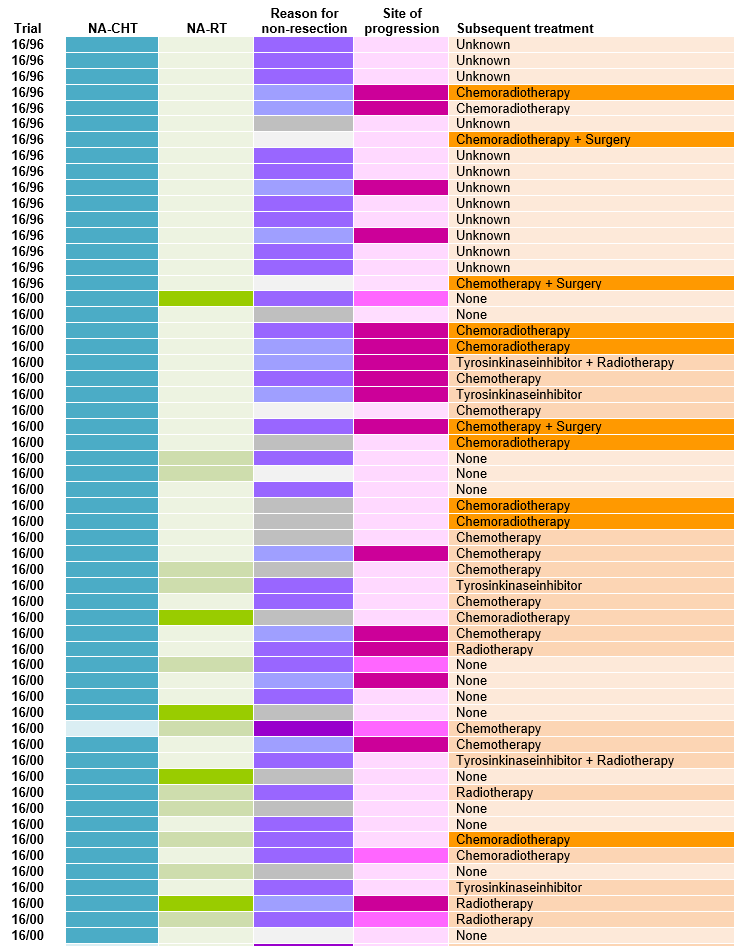

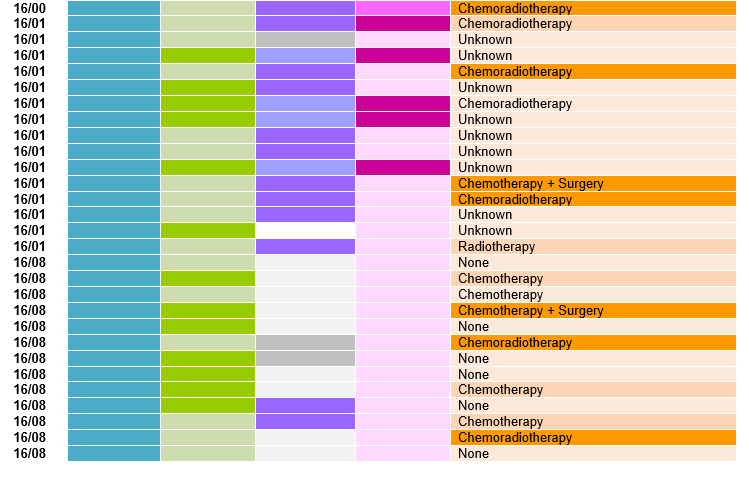


**Table S3.** Study treatment received, reasons for not undergoing definitive surgery, site of disease progression (if applicable), subsequent treatment, and subsequent treatment intent in SAKK 16/14. The first column (neoadjuvant chemotherapy, NA-CHT) describes whether the patient received neoadjuvant chemotherapy or not. The second column (neoadjuvant immunotherapy, NA-ICI) describes whether the patient received neoadjuvant immune checkpoint inhibitor or not. The third column summarizes the reasons for not undergoing definitive surgery (non-resection). The fourth column summarizes the site of progression (if known). The fifth column summarizes subsequent treatment following non-resection (if known).

**
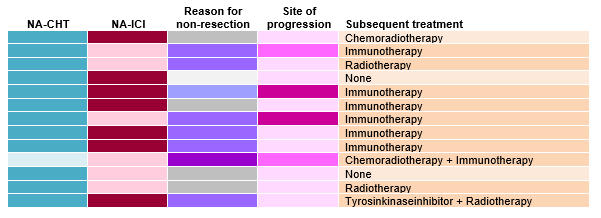
**

**
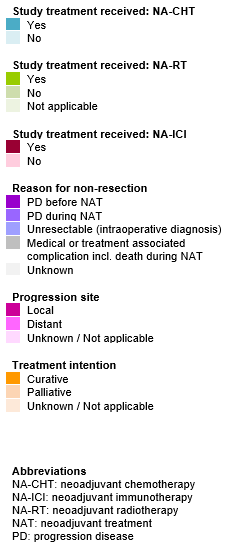
**

**Figure S1A and S1B.** Overall survival in all patients, as well as in those with and without definitive surgery, in the pooled analysis of non-ICI trials (SAKK 16/96, 16/00, 16/01, 16/08) (A) and in the SAKK 16/14 trial (B).

**
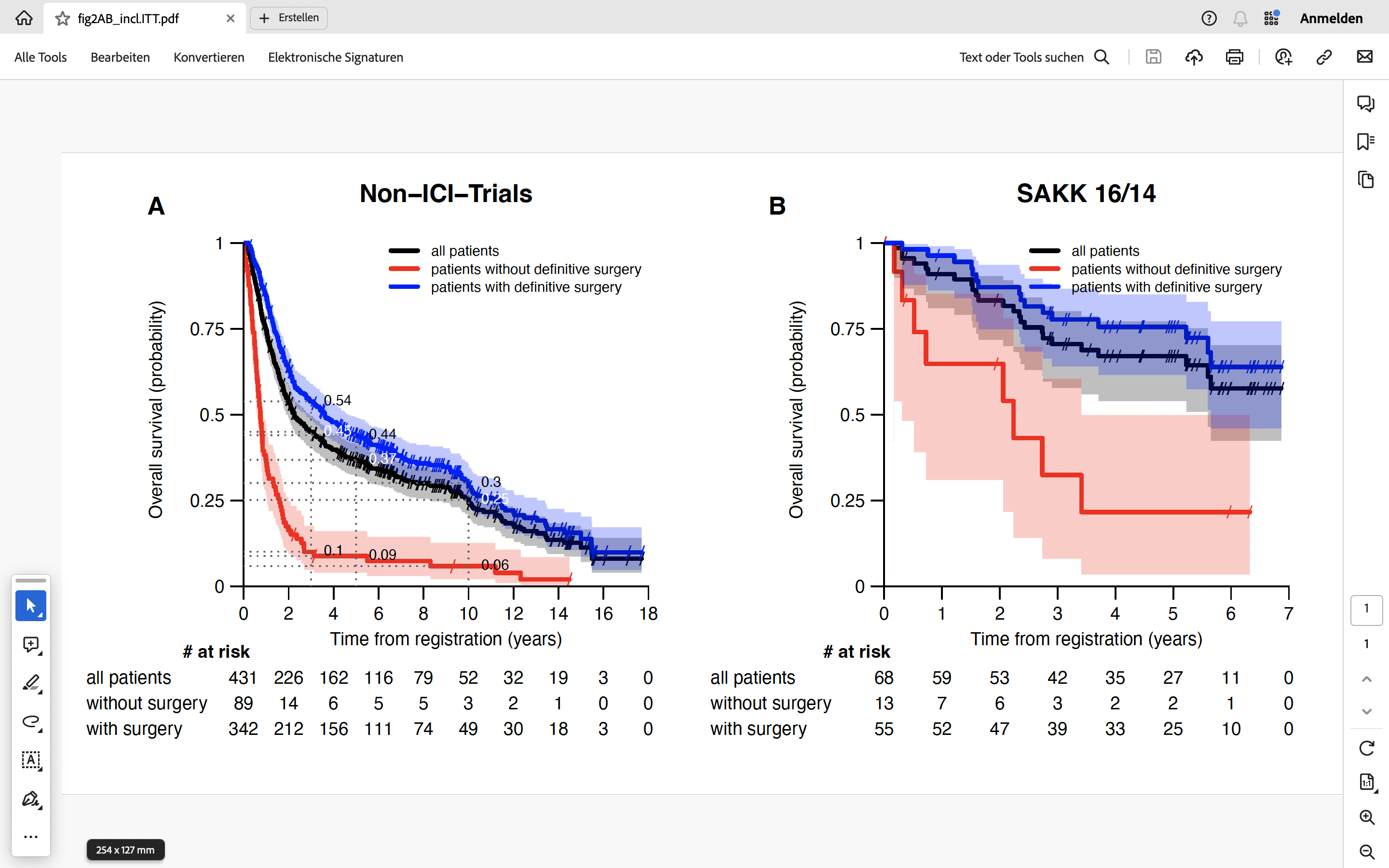
**

**Figure S2A and S2B.** Median overall survival (OS) according to resection status in the non-ICI trials (SAKK 16/96, 16/00, 16/01, 16/08) (A) and the SAKK trial 16/14 (B).
Cohort A includes patients without definitive surgery. Cohort B1 includes patients with R0 resection and Cohort B2 patients with R1/R2 resection.


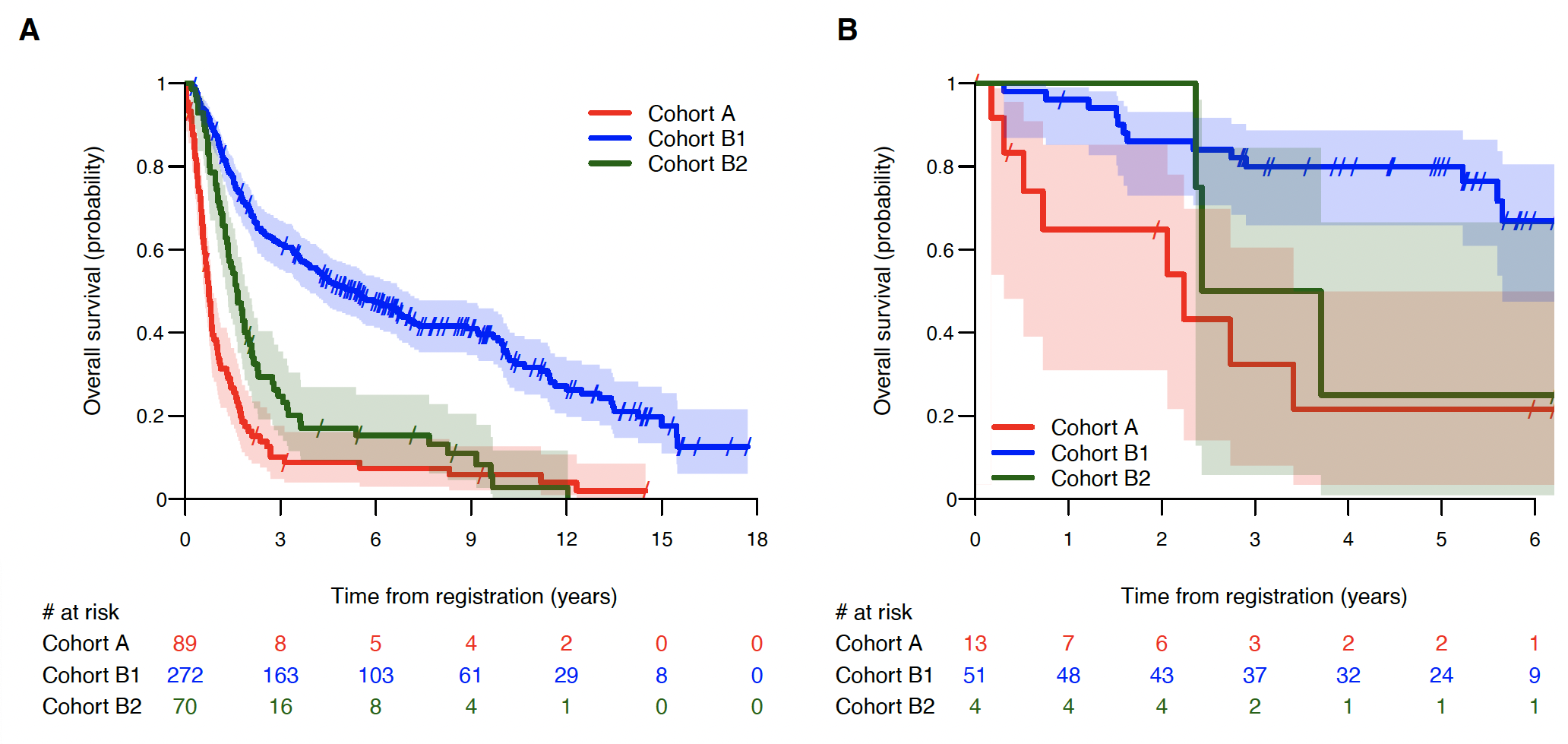

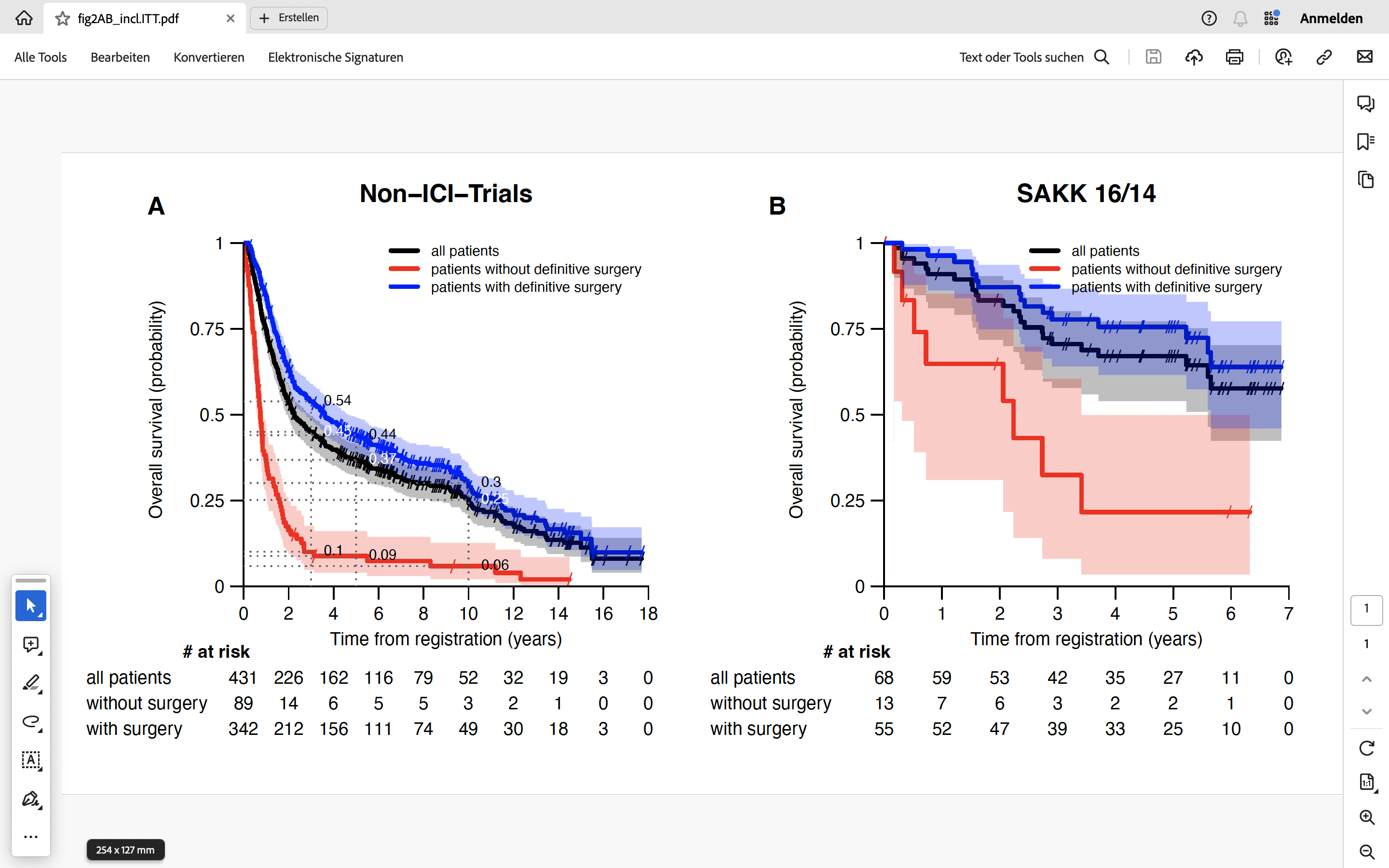


**Figure S3.** Overall survival in patients not undergoing definitive surgery (Cohort A), by subsequent treatment, irrespective of the intent behind the treatment.


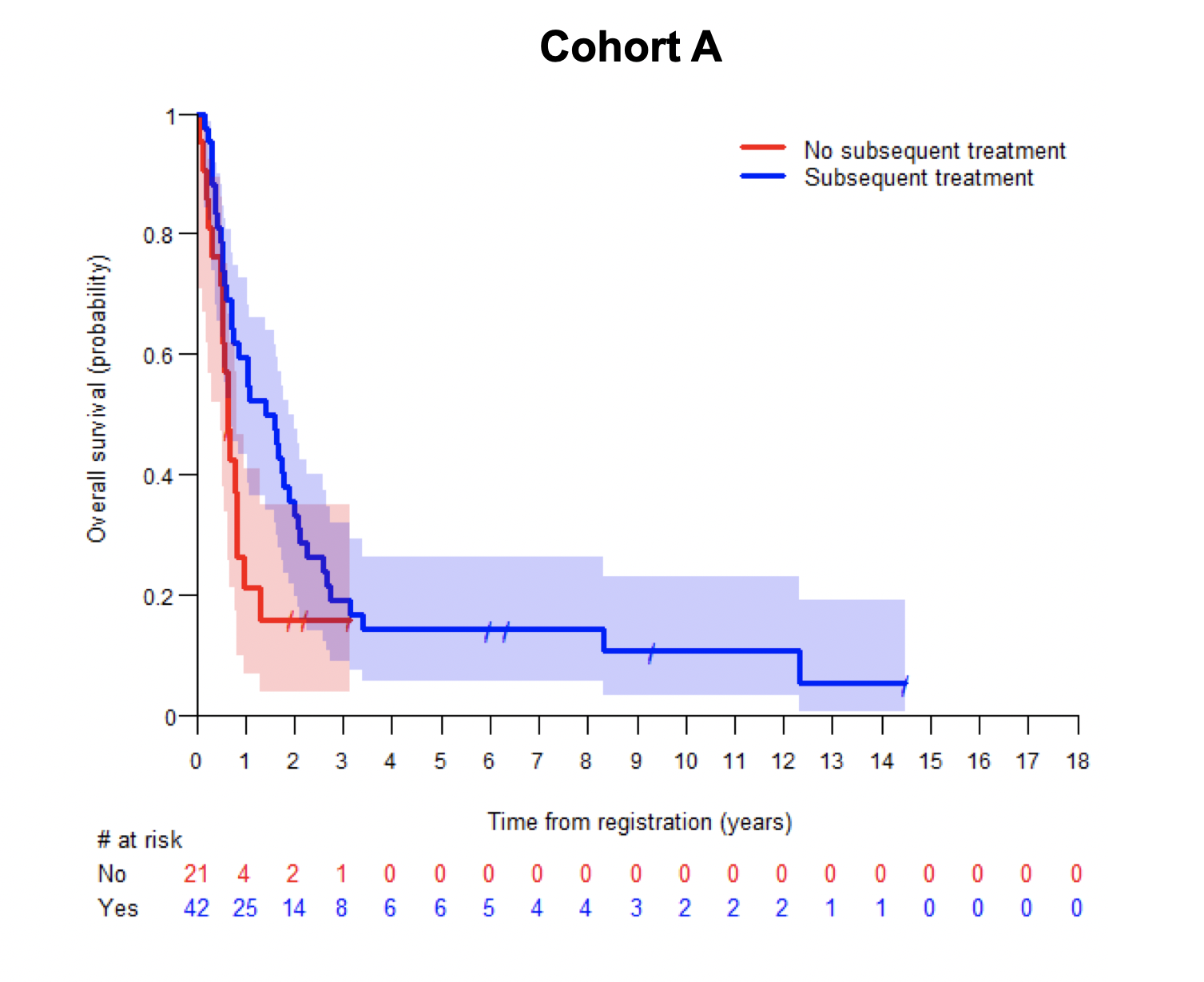

Supplement: Supplementary Tables 1-3 and Supplementary Figures 1-3 [file mmc1.docx]
